# Prevalence of Systemic Arterial Hypertension Diagnosed, Undiagnosed, and Uncontrolled in Elderly Population: SABE Study

**DOI:** 10.1155/2019/3671869

**Published:** 2019-09-03

**Authors:** Isabela Martins Oliveira, Yeda Aparecida de Oliveira Duarte, Dirce Maria Trevisan Zanetta

**Affiliations:** ^1^School of Public Health, University of Sao Paulo, São Paulo, Brazil; ^2^School of Nursing, University of Sao Paulo, São Paulo, Brazil

## Abstract

Systemic arterial hypertension is the most prevalent chronic noncommunicable disease among older people. This study aimed to estimate the prevalence of hypertension in the elderly and to analyze factors associated with diagnosed, undiagnosed, and uncontrolled hypertension. This is a cross-sectional study of data from the SABE study—Health, Well-Being, and Aging Survey—a multiple-cohort study, obtained in 2010, composed of a probabilistic sample representative of the population of the São Paulo city aged ≥60 years. Hypertension was self‐reported or defined by increased blood pressure. Multinomial regression assessed factors associated with diagnosis and lack of diagnosis of hypertension (reference: no hypertension), and logistic regression assessed factors associated with uncontrolled hypertension (reference: controlled). The prevalence of hypertension was 79.5%, and in 51% of individuals with the condition, hypertension was uncontrolled. Undiagnosed hypertension was associated with nonwhite skin color (OR: 1.89, CI: 1.11–3.19), being uninsured (OR: 1.77, CI: 1.04–3.03), overweight (OR: 2.38, CI: 1.09–5.19), higher education (OR: 0.46, CI: 0.22–1.94), and ≥1 chronic disease (OR: 0.28; CI: 0.13–0.58). Diagnosed hypertension was associated with age between 70 and 79 years (OR: 2.02, CI: 1.34–3.05), age ≥80 (OR: 2.73, CI: 1.72–4.31), nonwhite skin color (OR: 1.48, CI: 1.01–2.18), being uninsured (OR: 1.70, CI: 1.18–2.47), at least one medical consultation in the last year (OR: 1.86, CI: 1.06–3.25), obesity (OR: 2.50, CI: 1.61–3.88), and ≥1 chronic disease (OR: 2.81, CI: 1.94–4.08). Among those with hypertension, being uncontrolled was associated with widowhood (OR: 1.73, CI: 1.23–2.43), being uninsured (OR: 1.38, CI: 1.02–1.87), and female gender (OR: 0.61, CI: 0.43–0.87). The prevalence of hypertension was high in this population, and its diagnosis and control were associated with socioeconomic, demographic, and healthcare access factors.

## 1. Introduction

Systemic arterial hypertension is the most prevalent chronic noncommunicable disease (CNCD) in the world. More than 30% of adults are estimated to have the disease worldwide [1] with higher frequencies found with increasing age. However, the prevalence of the disease varies according to the location evaluated and measurement methods applied. A metanalysis evaluated studies of the older population in Brazil that used both self-reports and blood pressure (BP) measurements and estimated a prevalence of 68% of hypertension [2]. Since this age group also presents a higher prevalence of other CNCDs such as diabetes and cardiovascular diseases [3], the occurrence of hypertension or lack of control combined with other disorders can lead to a greater need for healthcare services, functional disabilities, poor quality of life, and greater risk of mortality in this population.

Populational studies that estimate the prevalence of hypertension are mostly directed towards the adult population and usually use self-reported data from their participants. The older population is represented by subsamples among the age groups investigated [4–6]. Studies that use objective measures and consider representative samples of this age group are necessary to understand the characteristics of this population and their healthcare needs. Health promotion and prevention strategies must be continuously planned, as the older people increases worldwide.

The aim of this study was to estimate the prevalence of hypertension in a representative sample of older individuals (≥60 years) residing in the urban area of the city of São Paulo, to describe their life and health characteristics, and to analyze factors associated with the occurrence of diagnosed, undiagnosed, and uncontrolled hypertension.

## 2. Materials and Methods

### 2.1. Population and Study Design

The present investigation is a cross-sectional study carried out using data collected in 2010 from the SABE study—Health, Well-Being, and Aging Survey, a multiple-cohort study. The SABE study began in 2000, with a probabilistic sample of older individuals (60 years of more) living in the city of São Paulo [7]. The elderly who had participated in 2000 (cohort A) were revaluated in 2006, in the second wave of the study. At this time, a new cohort of individuals between the age of 60 and 64 years was included in the study (cohort B), to maintain the representativeness of this age group in the sample. The third wave of the study took place in 2010, with a follow-up of the cohorts A and B and the inclusion of a new cohort of individuals between 60 and 64 years (cohort C). The selection of samples from the cohorts was conducted through the cluster sampling method over two stages, using proportionate size as a separation criterion: census sector and home. Samples were weighted according to the 2010 Census of the Brazilian Institute of Geography and Statistics to guarantee the representability of the older people residing in the municipality of São Paulo. Details on the sample design of the initial study are described in another publication [8].

### 2.2. Data Collection

Data were collected at participants' homes through a questionnaire applied by trained interviewers, through physical evaluations conducted by nutritionists with calibrated equipment to collect anthropometric measures, and through blood and urine collected by nurses. The Mini-Mental State Examination [9] was used to assess the cognitive level of participants at the beginning of the evaluation. If the individual did not reach the cutoff score of 12 points adapted for the SABE study [10], a proxy respondent was requested. Blood pressure was measured using a HEM-75CP Intellisense digital pressure monitor after the initial rest of 5 minutes. The individual under evaluation was asked to remain seated on a chair with back support, without crossing their legs, and with either their arm or elbow (preferentially the left one) supported on either a table or armrest. Three measurements were conducted, with a one-minute interval between each. The size of the blood pressure cuff used was chosen according to the brachial circumference of the participant. The first measurement of BP was excluded, and the mean of the two following measurements was calculated. The following parameters analyzed from the blood samples drawn were used in this study: serum creatinine, serum glucose (mg/dL), cholesterol and the HDL and LDL fractions (mg/dL), and triglycerides (mg/dL). Analyses were conducted at the laboratory of InCor (Heart Institute, School of Medicine, University of São Paulo), which is certified by ISO 9001.

### 2.3. Variables Used

Sociodemographic and economic characteristics were assessed (sex, age, educational level, marital status, and skin color), as were characteristics regarding use of and access to healthcare services (medical consultations over the last year, hospitalizations over the last year, use of services and hospitalizations specifically related to hypertension, and type of healthcare coverage) and health and functional performance aspects (self-evaluated health, cognitive decline, symptoms of depression, presence of chronic diseases, nutritional state, metabolic syndrome, presence of a caregiver, physical and mental quality of life, and treatment for hypertension).

#### 2.3.1. Definitions

*(1) Presence of Hypertension*. Older individuals were considered hypertensive when they either responded positively to the question “Has a doctor or nurse ever told you that you are hypertensive?” or were in use of antihypertensive medication or presented BP measurement ≥140/90 mmHg [11]. Hypertensive individuals were classified as (1) undiagnosed when there was no self-report of hypertension but mean measured BP was ≥140/90 mmHg, (2) controlled when individuals reported the disease and presented BP <140/90 mmHg, and (3) uncontrolled when individuals reported the disease and presented BP ≥140/90 mmHg. Older individuals were considered prehypertensive when either systolic blood pressure (SBP) was between 121 and 139 mmHg or diastolic arterial pressure (DBP) was between 81 and 89 mmHg, while individuals with normal BP were those that presented BP <140/90 mmHg and did not report any type of hypertension treatment [12].

*(2) Metabolic Syndrome*. It is defined by the presence of at least three of the following criteria [13]: waist circumference ≥90 cm for men and ≥80 cm for women, triglycerides ≥150 mg/dL, HDL cholesterol ≤40 mg/dL for men and ≤50 mg/dL for women, SBP ≥130 mmHg and DBP ≥85 mmHg, and fasting serum glucose ≥100 mg/dL [13].

*(3) Nutritional State*. The evaluation of nutritional state was conducted through the body mass index (BMI, kg/m^2^), following the PAHO criteria [14] for the elderly: underweight (BMI ≤ 23), normal weight (23 < BMI < 28), overweight (≥28 BMI < 30), and obese (BMI ≥ 30).

### 2.4. Statistical Analysis

Weighted proportions were estimated using sample weights according to the 2010 population census, with their respective confidence intervals for categorical variables, while weighted means and standard errors were used for continuous variables. Variables were compared by the chi-square (*χ*^2^) test with the Rao–Scott correction for complex samplings.

Multinomial logistic regression was used to assess factors associated with the diagnosis and lack of diagnosis of hypertension. Older individuals that were not hypertensive (normotensive and prehypertensive) were used as the reference category, and variables were inserted in blocks. The order and variables inserted in each block were based on studies on hypertension that used the same modelling technique [15–17] and were adapted according to the information available. The distal variables chosen covered the following sociodemographic and economic aspects: sex (male: reference and female), age group (60 to 69: reference, 70 to 79, and ≥80 years), years of schooling (0 to 3: reference, 4 to 7, and 8 or more), marital status (with partner: reference, without partner, and widow/widower), and skin color (white: reference and nonwhite). Nonwhite individuals were considered as those who did not self-report being white. This block was followed by the intermediate variables of use and access to healthcare services: medical consultations over the past 12 months (no: reference and yes) and healthcare coverage (has a health insurance: reference and public healthcare system users). The last block concerned aspects regarding the health of each individual: self-evaluated health (good: reference and bad), nutritional state (normal weight: reference, underweight, overweight, and obese), and chronic diseases (does not have any: reference and has one or more). Logistic regression (following the same previously described technique of inserting variables into blocks) was used to evaluate factors associated with uncontrolled arterial hypertension (outcome). For this model, older individuals with controlled hypertension composed the reference category. The final model of both regression models included variables with significance levels ≤0.05 through the Wald test and variables that were necessary for adjustments. Model goodness of fit was evaluated through the Hosmer–Lemeshow test. All analyses were conducted using the statistical software Stata 13.0 in the survey mode to allow the weighing of complex samplings.

## 3. Results

In 2010, a total of 1,344 older people were evaluated, with information on blood pressure (either measured or self-reported) unavailable for 111 people. The final sample of the study consisted of 1,233 participants, who represent 1,224,336 older people of the city of São Paulo. Among the individuals with no blood pressure information, there was a higher proportion of men (43.6% vs. 39.4% among evaluated individuals), people of age of 80 years or more (25.6% vs. 15.1% among evaluated individuals), and people with 8 years or more of formal education (31.5% vs. 26.3% among evaluated individuals). Most participants were women, aged 60 to 69 years, white, and with partners.

According to the criteria established, the prevalence of hypertension among this population was 79.5%. Hypertension was considered uncontrolled among 51% of individuals that reported the condition. Moreover, 9.1% presented high pressure levels without reporting a diagnosis of hypertension at the time of the evaluation. Although 20.4% of the older individuals in the city were not hypertensive, most were classified as prehypertensive (see Table 1).

In an additional analysis, 9.3% of hypertensive individuals presented BP levels that characterize isolated systolic hypertension (SBP ≥ 140 mmHg and DBP < 90 mmHg) [12]. Among these individuals, 74% were classified as having undiagnosed hypertension and 26% were classified as having uncontrolled hypertension.

As shown in Table 2, hypertensive individuals were more frequent in the age groups of 70 to 79 years and 80 or more than in the age group of 60 to 69 years, when compared to nonhypertensive participants. Hypertensive individuals were also more frequently nonwhite and with up to 3 years of formal education. The same occurred for the self-evaluation of bad health, poor quality of life regarding physical condition, obesity, presence of a caregiver, presence of all CNCDs evaluated (diabetes, cerebrovascular disease, and coronary disease), and metabolic syndrome. Regarding the use of and access to healthcare services, there was a higher frequency of hypertensive individuals using exclusively the public healthcare system and attending medical consultations over the last year.

Undiagnosed hypertension was observed more frequently in the individuals up to 69 years and that self-evaluated their health as good, when compared to the diagnosed individuals. There was a lower frequency of depressive symptoms, other chronic diseases such as diabetes and cerebrovascular disease, metabolic syndrome, and obesity in this group of older people with undiagnosed hypertension. Quality of life was also better in these individuals, with a lower frequency of QOL evaluated as bad, both in the physical and in the mental domain. Undiagnosed individuals had a lower frequency of medical consultations over the past year than the participants that received a diagnosis of hypertension (see Table 2).

The factors associated with the diagnosis and lack of diagnosis of hypertension (reference: normotensive) in the final multinomial logistic regression model are shown in Table 3. The odds that nonwhite older individuals had undiagnosed hypertension were 90% greater than those of white individuals. The odds of public healthcare system users were 77% higher than those of people with health insurance, and the odds of overweight people were 138% higher than those of eutrophic individuals. Older individuals with ≥8 years of formal education (reference: 0–3 years) and with at least one CNCD (reference: having none) presented lower odds for undiagnosed hypertension (a decrease of 54% and 72%, respectively).

Regarding the diagnosed hypertension (reference: normotensive), older individuals of the age groups of 70 to 79 years and 80 years or more presented odds 102 and 173% higher than those between 60 and 69 years, respectively. Nonwhite individuals presented odds 48% higher than white individuals. Public healthcare system users presented odds 70% higher than those with health insurance, people who had medical appointments in the last year presented odds 86% higher than those who did not have consultations, the obese ones presented odds 150% higher than the eutrophic ones, and older people with at least one CNCD had 181% higher odds than those without CNCD for diagnosed hypertension (see Table 3).

Among older people with previously diagnosed hypertension, a comparison was made between those with controlled and uncontrolled hypertension, as shown in Table 4. Uncontrolled hypertension was more frequent in widows/widowers and less frequent in single individuals and those living with a partner. A higher frequency of cognitive decline, diabetes, and metabolic syndrome was observed in individuals with uncontrolled hypertension. These individuals had a higher frequency of exclusive use of the public healthcare system and of healthcare services specific to hypertension when compared to those with controlled hypertension and a lowest frequency of medical consultations over the past year.

The factors associated with uncontrolled hypertension (reference: controlled hypertension) in the final multiple logistic regression model are shown in Table 5. Women presented 39% lower odds of having uncontrolled hypertension than men, while widows/widowers (reference: having a partner) and public healthcare system users (reference: having health insurance) presented 73% and 38% higher odds, respectively.

## 4. Discussion

This population-based study with a representative sample of the older population (≥60 years) assessed the prevalence of hypertension, by self-report, use of antihypertensive medication, and blood pressure measurement performed according to the JCN criteria [11]. The striking high prevalence of hypertension found in the older population of São Paulo was similar to the prevalence of other population-based studies in Brazil in this age group, when a similar methodology was used: 75.6% among the elderly in the northeast region of Brazil [18] and about 85% in the elderly in the southern region of the country [19, 20]. In the Brazilian Legal Amazon region, the prevalence among older people was 67.4%, lower than that in other parts of the country [21].

Half of those who had the diagnosis had uncontrolled hypertension, and approximately 10% of the older people living in the city presented high blood pressure without knowing it. Moreover, among the individuals without the disease, more than half were classified as having prehypertension. The disease was shown to be prevalent with increasing age, as was the lack of control. The prevalence of hypertension among people aged 80 or more was even higher, as was described in studies [18, 19]. Data from the Brazilian National Household Sample Survey (Pesquisa Nacional de Amostra por Domicílios (PNAD)) that analyzed the prevalence of hypertension from 1998 to 2008 also showed a significant increase in the occurrence of hypertension in the 80+ group over time [22].

Studies evaluating self-reported hypertension reported a lower prevalence of around 60% among older people living in Brazilian capitals [23, 24]. In the first two waves of the SABE study, in 2000 and 2006, when blood pressure was not measured, the prevalence of self-reported hypertension was 53.1% and 62.7%, respectively. Considering only self-reported disease, we found a prevalence of 67.4% in 2010, showing an increase with time. When blood pressure and use of antihypertensive medication were also considered, the prevalence was 79.5%. The difference in prevalence obtained when comparing objective measurements and self-reports of hypertension is widely recognized in the literature [19, 23, 25, 26]. Data from the World Health Organization's Study on Global Aging and Adult Health (SAGE) show that, in South Africa, the prevalence of hypertension among the elderly is 77.9%. However, less than 40% of those evaluated had previous diagnosis. The same study shows that, in low-income countries, this difference is even higher. In Ghana, only 23% of hypertensive individuals reported the diagnosis of hypertension [27].

Another population-based study conducted in the United States showed that 64.9% of elderly Americans had hypertension, but 12.5% of them did not self-report hypertension in the evaluation [28, 29].

Hypertensive older individuals presented worse health indicators, with higher frequencies of self-evaluated bad health, poor quality of life, need for a caregiver, and other chronic diseases than those without the disease. These characteristics corroborate with other studies that show the relationship of presence of hypertension and poorer health condition, even in subjective aspects such as the self-evaluation of quality of life, since the disease requires more frequent visits to healthcare services, continuous use of medication, and can cause symptoms and discomfort, which can negatively influence the perception of the individuals regarding their own health [30].

Individuals that were not hypertensive were more frequently observed between the age of 60 and 69 years, white, with a higher educational level (≥8 years), and with a lower prevalence of CNCDs and other risk factors for hypertension such as being overweight or obese than those being hypertensive. However, despite the lower frequency of risk factors, more than half of the nonhypertensive older individuals did in fact present blood pressure measurements that characterize prehypertension. The risk of prehypertensive women and men aged between 45 and 64 years becoming hypertensive within four years was shown to exceed three times that of normotensive individuals [31], reinforcing the importance of monitoring the health condition of these individuals to either avoid or postpone the progression to hypertension.

The positive relationship found between old age and hypertension (both diagnosed and undiagnosed) agrees with that reported in several other studies [32, 33]. Typical alterations caused by aging such as arterial stiffness, endothelial disorder, decreased renal function, and increased load of related diseases make the elderly more prone to the development of hypertension [34]. Nonwhite older individuals presented higher odds of being affected by hypertension. The black population due to several factors, which include genetic alterations, is more prone to have hypertension [35, 36]. In the present study, it was not possible to analyze the relationship between hypertension and black skin color. The reports of skin color (except for whites) were grouped into only one category because of the low frequency of some categories. Also, there is a lack of correspondence between race/skin color categories used in Brazilian studies and those used in international studies, as Brazil has a highly mixed population.

Having more years of formal education was a protective factor for undiagnosed hypertension. In general, education tends to present a positive association with better health outcomes. A study carried out in the city of Florianópolis showed that the low educational level is associated with increased SBP and increased odds of hypertension among older people [37]. The hypothesis is that individuals with higher educational levels are more knowledgeable about health and, thus, seek healthcare services more often. In addition, a higher educational level is associated with a higher socioeconomic level, which is another indicator for higher access to healthcare services [38, 39]. In this study, we used the educational level as an indicator for the socioeconomic status. Another variable that reflects the socioeconomic level is the usage of the public healthcare system. Brazil has adopted a universal healthcare system since 1988, called the Brazilian Unified Health System (Sistema Único de Saúde (SUS)). It offers healthcare services that are free of charge at point of delivery. SUS is mainly used by lower-income and lower-middle-income population, but it is available to every citizen in the country, even the ones who pay for private healthcare insurance. In Brazil, private health insurance is used by 28% of the population [40]. The higher odds of having hypertension (diagnosed and undiagnosed) among older people who use the public healthcare system show the influence that socioeconomic characteristics have on both the occurrence of hypertension and its diagnosis.

The presence of one or more CNCDs was positively associated with the diagnosis for hypertension, as shown in the literature [41, 42]. Individuals who have CNCD need frequent healthcare follow-up and, therefore, are more likely to have their blood pressure measured. In fact, the hypertensive ones in the present study reported more medical consultations over the last year than the normotensive ones.

Overweight individuals presented higher odds of having the undiagnosed disease, while the obese ones presented higher odds of being diagnosed. The association between weight and hypertension among older people has been previously shown [43, 44]. The mechanisms that associate excess weight with hypertension include hyperactivity of the sympathetic nervous system, related especially to the buildup of visceral abdominal fat. The increase of this type of fat is associated with an increase of inflammatory mediators, oxidative stress, and decreased endothelial vasodilatation [45].

The high prevalence of chronic diseases and their inadequate control have important implications for the healthcare system. This happens especially in countries like Brazil, which has undergone an accelerated process of demographic transition, with a rapid increase of older people, leading to a healthcare system that was unprepared to meet new demands so quickly [46].

National measures for the prevention and treatment of CNCDs and risk factors have been implemented in Brazil, such as strategies to reduce smoking, increased taxes on tobacco and alcoholic beverages, distribution of free medication for diabetes, hypertension, and other diseases, creation of a national registry to follow up public healthcare system users who are diabetic and hypertensive, and the training of public healthcare professionals towards tracking and caring for chronic diseases [47–49]. Despite these strategies, particularly those specific to the public healthcare system users, there was a significant association between using the public healthcare system and having uncontrolled hypertension in our study. It has been shown that health insurance users present higher rates of diagnosis and controlled hypertension [50, 51]. In Brazil, results from the ELSI study also showed a positive association between adequate BP control and the use of private healthcare [52]. As users of the public healthcare system in Brazil are mostly individuals with worse socioeconomic conditions, these results also suggest some influence of socioeconomic factors on the control of chronic diseases. However, public healthcare system users reported a greater number of medical consultations over the past year, suggesting that even with access to healthcare, there are other factors that hinder control over the disease. Adequate control, especially among older people, can prevent more serious complications from this condition, such as strokes and kidney disease.

The high frequency of uncontrolled hypertension is consistent with that reported in the literature [18, 27]. A systematic review of Brazilian studies on hypertension control (for all age groups) showed that control rates range from 10% to 57.6% in the country. However, in this review, only a few studies in the northern and northeastern regions were found. Three studies with a population-based sample that evaluated only older people were found in the review, all in the southeast region, and the control ranged from 27% to 44.6% [53]. Recent data from the ELSI study, considering older people residing in 70 Brazilian cities, showed that only 51% had BP controlled [52].

Other factors associated with lack of control of hypertension were sex and marital status. Women presented lower odds of uncontrolled disease. Despite the increased risk of hypertension and other cardiovascular conditions among older women due to menopause and reduced protective factor from estrogen [54], studies have shown that women and individuals above the age of 64 years seek out preventive healthcare services more often and follow up on their condition at reference centers [55, 56]. In an additional analysis, data from the present study showed that older women had a higher frequency of medical appointments in the last year than men. Our findings on the greater frequency of use of healthcare services among women are consistent with those reported in the literature [56]. BP control was less frequent among men, which may be one of the consequences of the poor usage of healthcare services in this group. Specific interventions for men in healthcare settings can strengthen the relationship between the healthcare service and the male user and be beneficial for the control of various diseases, including hypertension [57].

The uncontrolled hypertension was more frequent in widows/widowers. Van Rossum et al. [58] emphasize the role of the spouse in the management of hypertension, especially if the hypertensive individual demands family support to change their lifestyle as part of nonpharmacological treatment for hypertension.

Elderly with controlled and uncontrolled hypertension presented similar frequencies of treatment, although individual treatments were reported more by elderly with controlled hypertension and the combined treatment (pharmacological and nonpharmacological methods) by those with uncontrolled hypertension. This may indicate that uncontrolled hypertension is more related to greater disease severity and difficulties in blood pressure control than to lack of treatment. For this study, only reports of treatment used were assessed, since the study protocol does not allow evaluations of other aspects such as adherence to the treatment prescribed by healthcare professionals.

The present study has some limitations: The blood pressure measurement at one time is not adequate for the diagnosis of hypertension. However, other epidemiological studies with one-time measurements have shown important predictive power for hypertension and other cardiovascular diseases [59, 60]. In the present study, the measurements were performed at the residence of the participant, after initial rest. The first measure was not used, and the average of the last two was taken as the final result. These measures may have minimized the possible “white coat” effect. Approximately 10% of the sample evaluated in the SABE study in 2010 had no information related to hypertension and therefore did not compose the final sample of the present study. Although the sample loss was not large, it is not possible to rule out the possibility of bias in the population estimates. In the nonevaluated elderly, compared to those who composed the final sample, there was a higher frequency of men, older people with higher education, and those aged 80 years or more. Gender was not associated with hypertension in this study, but higher education and increased age had lower and higher frequencies of hypertension, respectively, than elderly with lower education and decreased age. On the contrary, epidemiological studies that use objective blood pressure measurement in representative samples are important to obtain data on the impact of diseases on populations.

## 5. Conclusions

In conclusion, the prevalence of hypertension was very high, mostly in older age groups, in nonwhite individuals, and in individuals with low education levels and worse health conditions. Hypertension was associated with demographic and socioeconomic factors, health conditions, and access to healthcare services. Older people with more years of schooling and other chronic diseases were less likely to be undiagnosed. Although access to healthcare was associated with the occurrence of hypertension and lack of control, there was no evidence of lack of access to healthcare services (public and private) in this population. The results may contribute to the formulation of appropriate public policies for this age group of the population.

## Figures and Tables

**Table 1:** Classification of the older people living in São Paulo in 2010, according to blood pressure measures and diagnosis of hypertension.

	*n*	Weighted population^*∗*^	Frequency (%) (95% CI)	Mean SBP (SE)	Mean DBP (SE)
Nonhypertensive	**235**	**250.274**	**20.4 (20.3-20.4)**	**122.2 (0.7)**	**74.8 (0.6)**
Normotensive	98	100.496	8.2 (8.1-8.2)	112.1 (0.8)	70.2 (0.7)
Prehypertensive	137	149.777	12.2 (12.1-12.2)	129.0 (0.5)	78.0 (0.7)
Hypertensive	**998**	**974.361**	**79.5 (79.3–79.6)**	**145.6 (0.7)**	**81.9 (0.4)**
Undiagnosed	110	111.596	9.1 (9.0-9.1)	153.8 (1.3)	87.4 (0.9)
Controlled	443	421.197	34.3 (34.1–34.4)	126.4 (0.5)	74.9 (0.4)
Uncontrolled	445	441.567	36.0 (35.8–36.1)	161.9 (0.9)	87.3 (0.6)

^*∗*^Results presented were weighted to be representative of the older population of São Paulo based on the 2010 Census of Brazil; SBP: systolic blood pressure (mmHg); DBP: diastolic blood pressure (mmHg). SBP and DBP are represented as the mean of the last two measures (standard error).

**Table 2:** Life and health characteristics of older people living in São Paulo in 2010, according to blood pressure measurement and diagnosis of hypertension.

	General distribution (*n* = 1224635)^*∗*^	Normotensive			Hypertensive			*p* ^a^	*p* ^b^
		Total (*n* = 250274)	BP < 140/90 (*n* = 100496)	Prehypertensive (*n* = 149777)	Total (*n* = 974361)	Diagnosed (*n* = 862764)	Undiagnosed (*n* = 111596)		
	%	%	%	%	%	%	%		
Sex									
Men	39.39	42.36	31.04	49.95	38.62	37.55	46.92	0.352	0.08
Women	60.61	57.64	68.96	50.05	61.38	62.45	53.08		
Age									
60–69	55.1	64.3	57.4	69.1	52.6	50.8	66.5	0.003	0.005
70–79	29.8	25.13	30.8	21.3	31,0	31.97	23.4		
≥80	15.14	10.51	11.9	9.5	16.33	17.15	9.9		
Marital status									
With partner	55.43	58.25	54.2	60.9	54.7	55.1	25,0	0.1105	0.7775
Single	13.11	16.09	16.2	15.9	12.3	11.3	20.7		
Widow/widower	31.46	25.66	29.4	23.1	32.9	33.6	27.2		
Skin color									
White	58.18	66.91	69.41	65.21	55.95	56.92	48.47	0.005	0.1217
Nonwhite	41.82	33.0	30.5	34.79	44.0	43.08	51.53		
Years of schooling									
0–3	36.11	26.86	27.2	26.6	38.49	38.17	40.9	0.0002	0.7775
4–7	37.58	35.93	43.2	31.0	38.0	37.96	38.2		
≥8	26.31	37.21	29.5	42.3	23.51	23.87	20.7		
Self-rated health									
Good	50.97	59.54	56.8	61.3	46.34	43.62	67.3	0.0011	<0.001
Bad	49.03	40.46	43.5	38.6	53.66	56.38	32.6		
Cognitive impairment	10.12	7.56	8.4	6.9	10.78	11.17	7.8	0.114	0.2774
Depressive symptoms	19.76	16.87	16.8	16.9	20.5	21.5	12.6	0.266	0.038
Caregiver	20.7	14.07	20.5	9.7	22.48	23.17	17.1	0.004	0.1739
Diabetes	25.99	10.98	11.9	10.3	29.85	32.55	8.9	<0.001	<0.001
Coronary disease	23.39	12.4	15.6	10.1	26.22	29.49	1,0	<0.001	<0.001
Cerebrovascular disease	7.11	2.91	3.7	2.3	8.18	9.21	0.3	0.008	<0.001
Metabolic syndrome	50.96	35.02	23.9	42.6	55.07	56.45	44.4	<0.001	0.033
Nutritional status									
Underweight	13.12	17.65	21.5	15.0	11.95	11.35	16.4	0.0006	0.0415
Eutrophic	39.28	47.3	40.7	51.5	37.21	36.67	41.3		
Overweight	15.4	13.85	12.9	20.0	15.8	15.24	16.5		
Obesity	32.19	21.2	22.4	20.3	35.04	36.73	22.2		
Poor QoL: physical domain	45.72	36.59	40.4	34.0	48.1	50.1	32.2	0.0041	0.0013
Poor QoL: mental domain	48.94	43.79	43.8	43.7	50.28	51.63	39.6	0.114	0.0327
Insurance information									
Uninsured	43.73	45.71	45.7	45.7	58.98	58.03	66.3	0.0009	0.1283
Insured	56.27	54.29	54.3	54.2	41.02	41.97	33.7		
Had a medical consultation in the last year	87.81	83.1	86.1	81.0	89.02	91.16	72.5	0.024	<0.001
Hospitalized in the last year	10.93	7.48	8.9	6.4	11.82	12.1	9.6	0.0729	0.542

^*∗*^Results presented were weighted to be representative of the older population of São Paulo based on the 2010 Census of Brazil; ^a^comparison of normotensive versus hypertensive; ^b^comparison of diagnosed versus undiagnosed hypertension; QoL = quality of life.

**Table 3:** Life and health characteristics of diagnosed hypertensive older people living in São Paulo in 2010.

	Total (*n* = 862764)^*∗*^	Controlled (*n* = 421197)	Uncontrolled (*n* = 441567)	*p*
	%	%	%
Sex				
Men	37.55	34.3	40.6	0.086
Women	62.45	65.6	59.3	
Age				
60–69	50.89	50.2	51.4	0.4906
70–79	31.97	33.6	30.3	
≥80	17.15	16,0	18.1	
Marital status				
With partner	55.08	57.8	52.4	0.0481
Single	11.3	12.7	9.9	
Widow/widower	33.62	29.3	37.6	
Skin color				
White	56.92	57.14	56.7	0.9038
Nonwhite	43.08	42.86	43.3	
Years of schooling				
0–3	38.17	35.9	40.3	0.4647
4–7	37.96	39,0	36.8	
≥8	23.87	25.1	22.6	
Self-rated health				
Good	43.62	43.4	43.8	0.9074
Bad	56.38	56.6	56.1	
Cognitive impairment	11.17	8.6	13.5	0.0155
Depressive symptoms	21.5	20.9	22,0	0.7022
Caregiver	23.17	20.5	25.6	0.0753
Diabetes	32.55	28.8	36.0	0.0381
Coronary disease	29.49	32.2	26.8	0.1045
Cerebrovascular disease	9.21	7.3	10.9	0.0706
Metabolic syndrome	56.45	46.3	66.0	<0.001
Nutritional status				
Underweight	11.35	12.1	10.6	0.6573
Eutrophic	36.67	35.2	38.1	
Overweight	15.24	13.9	15.4	
Obesity	36.73	37.2	32.1	
Poor QoL: physical domain	49.9	49.2	50.9	0.6481
Poor QoL: mental domain	48.37	55.2	48.1	0.0608
Insurance information				
Uninsured	58.03	53.7	62.1	0.0208
Insured	41.97	46.2	37.8	
Had a medical consultation in the last year	91.16	93.7	88.6	0.0174
Hospitalized in the last year	11.82	11.5	12.6	0.6539
Use of specific healthcare services for hypertension care	26.29	22.82	29.45	0.0416
Hospitalizations due to hypertension	13.29	13.8	12.7	0.6833
Type of treatment				
Only pharmacological	54.8	57.25	52.18	0.4193
Only nonpharmacological	2.06	2.37	1.72	
Both	43.15	40.38	46.11	

^*∗*^Results presented were weighted to be representative of the older population of São Paulo based on the 2010 Census of Brazil; QoL = quality of life.

**Table 4:** Factors associated with diagnosed and undiagnosed hypertension in older people living in São Paulo in 2010.

	Undiagnosed (*n* = 111596)^*∗*^			Diagnosed (*n* = 862764)^*∗*^		
	OR^a^	95% CI	*p*	OR^a^	95% CI	*p*
Age						
60–69	1			1		
70–79	1.12	(0.60–2.12)	0.706	**2.02**	**(1.34–3.05)**	**0.001**
≥80	1.07	(0.53–2.17)	0.830	**2.73**	**(1.72–4.31)**	**<0.001**
Years of schooling						
0–3	1			1		
4–7	0.78	(0.43–1.39)	0.405	0.95	(0.63–1.41)	0.804
≥8	**0.46**	**(0.22–0.94)**	**0.035**	0.75	(0.47–1.20)	0.237
Skin color						
White	1			1		
Nonwhite	**1.89**	**(1.11–3.19)**	**0.017**	**1.48**	**(1.01–2.18)**	**0.042**
Insurance information						
Uninsured	1			1		
Insured	**1.77**	**(1.04–3.03)**	**0.035**	**1.70**	**(1.18–2.47)**	**0.005**
Medical consultation in the last year						
No	1			**1**		
Yes	0.56	(0.30–1.05)	0.075	**1.86**	**(1.06–3.25)**	**0.029**
Nutritional status						
Underweight	1			1		
Eutrophic	0.97	(0.48–1.95)	0.940	0.76	(0.47–1.24)	0.281
Overweight	**2.38**	**(1.09–5.19)**	**0.029**	1.43	(0.82–2.51)	0.202
Obesity	1.40	(0.72–2.72)	0.317	**2.50**	**(1.61–3.88)**	**<0.001**
NCDs						
None	1			1		
≥1	**0.28**	**(0.13–0.58)**	**0.001**	**2.81**	**(1.94–4.08)**	**<0.001**

Reference category: nonhypertensive. ^*∗*^Results presented were weighted to be representative of the older population of São Paulo based on the 2010 Census of Brazil; ^a^model adjusted by sex; NCDs: noncommunicable diseases.

**Table 5:** Factors associated with uncontrolled hypertension in older people living in São Paulo in 2010.

	Adjusted OR^*∗*^ (95% CI)	*p*
Sex		
Men	1	
Women	**0.61 (0.43–0.87)**	**0.007**
Marital status		
With partner	1	
Single	0.91 (0.55–1.51)	0.739
Widow/widower	**1.73 (1.23–2.43)**	**0.002**
Insurance information		
Uninsured	1	
Insured	**1.38 (1.02–1.87)**	**0.036**

Reference category: older people with controlled hypertension; ^*∗*^adjusted by nutritional status and NCDs.

## Data Availability

The data used to support the findings of this study are available from the corresponding author upon request.
